# Contemporary and dynamic effects of socio-economic factors on physical (in)activity: Does intensity matter?

**DOI:** 10.3389/fpubh.2022.1016353

**Published:** 2022-10-06

**Authors:** Christian M. García-Witulski

**Affiliations:** ^1^Centro de Desarrollo Humano Sostenible, Facultad de Ciencias Económicas, Pontificia Universidad Católica Argentina, Ciudad Autónoma de Buenos Aires, Buenos Aires, Argentina; ^2^Universidad Espíritu Santo, Guayaquil, Ecuador

**Keywords:** physical activity, socio-economic factors, dynamic effects, intensity, health

## Abstract

**Objective:**

This paper identifies varying contemporary and dynamic effects of socio-economic factors on individuals' decisions to allocate their time to physical activities when the intensity of these activities comes into play.

**Methods:**

Based on repeated cross-sectional data sourced from the Argentinean National Risk Factor Surveys of 2005, 2009, and 2013, we developed 18 fictitious cohorts to set up a pseudo panel. To address endogeneity problems, four econometric specifications were estimated: OLS, Heckman two-stage model, fixed- and random-effects models.

**Results:**

We find that changes in the opportunity cost of time are highly significant and provide shifts in individuals' decisions regarding the allocation of their time to physical activity consumption. When considering the intensity at which physical activities are consumed, increased income impacts less, suggesting that individuals faced with a wage increase reduce the time of consumption but increase its intensity. An interesting finding is that employed people consume more physical activity than inactive individuals. This indicates that the substitution effect produced by an increase in the wage rate is less than the income effect. Additionally, the increase in the coefficient of employed persons is greater when the intensity factor is considered, indicating that for employed individuals a trade-off between time and intensity is generated. We also found that higher levels of education increase the probability of participating in physical activities, but decrease the time spent in such activities. Furthermore, there are heterogeneous impacts on physical activity consumption between males and females, which can be observed in the strong effect of household production for women with at least one child. Finally, such impacts remain in a variety of estimated specifications.

**Conclusions:**

These results may be useful in order to suggest some tools for the design of interventions that are aimed at increasing participation in physical activities.

## 1. Introduction

Insufficient physical activity has been maintained for decades as an internationally important risk factor that crosses all ages and genders. Recently, a study conducted in 168 countries (on about 1.9 million people) estimated the prevalence of insufficient physical activity at the global level is 27.5% (1).

World Health Organization (WHO) guidelines on physical activity recommend for adults aged 18–64 years at least 150–300 min of moderate-intensity physical activity, or at least 75–150 min of vigorous-intensity physical activity throughout the week to maintain good health (2). In turn, there is strong scientific evidence linking physical inactivity to so-called non-communicable diseases (NCDs), including coronary heart disease, type 2 diabetes, and different types of cancer (3). However, to recognize the problem's seriousness, not only this association but also the global burden of physical inactivity on NCDs should be analyzed. For example, a study by Park et al. (4) indicates physical inactivity in the adult population causes about 6% of the burden of coronary heart disease, 7% of type 2 diabetes, and 10% of both breast and colon cancer.

Furthermore, through the rise in mortality *via* NCDs, physical inactivity creates a major burden on the economy and public health systems. Ding et al. (5) have quantified direct medical costs, lost productivity, and disability-adjusted life-years attributable to physical inactivity due to coronary heart disease, type 2 diabetes, and breast and colon cancer for 142 countries (about 93% of the global population). These estimates state that direct medical costs amount to USD 53.8 billion and lost productivity costs are USD 13.7 billion, while 13.4 million years adjusted for disability are lost.

Acknowledging this issue, there was the signing of the Bangkok Declaration on physical activity for global health and sustainable development in 2016, which strongly emphasizes the urgency of taking measures to promote physical inactivity reduction and of linking it to the fulfillment of certain objectives of the sustainable development agenda. In the meantime, the WHO launched in 2018 an action plan aimed at reducing by 15% (using 2016 as the baseline year) the global physical inactivity prevalence in adults and adolescents by the year 2030 (6).

In Argentina, the second-highest-populated country in South America, the situation does not differ from the overall scenario. The last three National Risk Factor Surveys (NRFSs) show that low physical activity prevalence among the population has been increasing in the last 15 years. The first NRFS conducted in 2005 showed a physical inactivity prevalence of 46.2%, which increased in just five years to 54.9%, concluding the latest estimates for 2013 at 55.1% (7). In this country, it has been calculated that physical activity could account for about 4% of deaths from non-communicable diseases and almost 3% of deaths from all causes (8). In addition, it has been estimated that the annual indirect economic costs due to cardiovascular deaths associated with physical inactivity reach 1,197 million international dollars (9).

Although several studies analyze the impact of socio-economic factors on physical inactivity using national surveys, two problems can be identified from the methodological point of view. In most studies, endogeneity problems are likely to occur through reverse causality between physical activity and health (10) as well as between physical activity and income (11), which are only discussed in these documents (the latter being the only one that applies the instrumental variables method, using the unemployment rate as an income instrument). Likewise, most of the papers use logit or probit models [e.g., (12)] in which only the number of individuals who perform physical activity at a given time is identified. Consequently, if the intensity factor plays a preponderant role in improving health, then probably by not considering the intensity at which said physical activity is carried out, it cannot be known whether the coefficients of the variables included as relevant in the estimates remain constant or change.

This work introduces two novel contributions. The first is the use of Deaton's technique for the construction of a pseudo-panel by developing a series of fictitious cohorts using the NRFSs 2005, 2009, and 2013 (13). This method has been previously used to examine temporary variations on cigarette consumption (14) and to explore changes in fruit and vegetable consumption in children and adolescents through the years (15). In this way, the problem of not having a longitudinal survey is solved, a situation that occurs in most developing countries because these types of surveys are much more expensive than cross-sectional surveys. Specifically, using this technique allows estimating the future rate of relative variation associated with physical activity and recognizing the dynamic nature of the problem. That is, certain factors like income, BMI, and education need time to materialize in the individual's behavior, and this cannot be captured using cross-sectional data. The second contribution is to capture how various factors influence not only physical activity consumption but also the intensity of such consumption. Most studies use logistic models where the dependent variable takes the value of 1 if individuals perform intense or moderate activities and 0 for the rest. However, these models do not provide information about whether the effects of various factors considered produce divergences on an individual's decision to allocate their time for physical activities when the level of intensity of said activity comes into play. This is particularly important since more intense physical activities achieve a more significant decrease in the probability of cardiovascular risk than less intense activities (16). In order to incorporate the intensity of physical activity consumption into the outcome variable, the metabolic equivalent of task (METs) is used as a weighting factor, which captures the intensity impact on time allocated to the physical activity consumption.

Regarding the structure of this work, Section 2 describes the data used. Section 3 explains the econometric approach. Section 4 shows the results obtained, and finally, Section 5 concludes the work and discusses its limitations and possible extensions.

## 2. Data

### 2.1. Study design and population

Data from the NRFSs (2005, 2009, 2013) were used in this paper. These are face-to-face surveys nationally conducted using a multi-stage probability sample using the national urban sampling framework (NUSF). The target population comprises persons aged 18 years and older living in private households in the country in urban areas with at least 5,000 inhabitants. In the NRFS 2005, 46,308 households were surveyable, and 41,392 households responded, representing a total population of 22,935,297. For the NRFS 2009, the original sample was 42,188 households, and 34,732 households responded, representing a total population of 24,434,595 people. Finally, in the NRFS 2013, from the original sample of 46,555 households, a response was obtained from 32,365 households representing 25,777,587 people. In the three NRFSs, a household questionnaire was applied to survey demographic, socio-economic, and residential aspects, and an individual questionnaire to collect data on one's general work and health situation was applied to a single, randomly selected individual among household members.

These surveys constitute a rich resource of information to explore the factors associated with physical activity, and although no studies have been found that have used them for this purpose, they have been used in previous studies such as Fleischer et al. (17) to study the relationship between risk factors for chronic diseases and socio-economic status as well as Linetzky et al. (18) and Monteverde et al. (19) to assess the relationship between being overweight or obese and socio-economic position.

#### 2.1.1. Physical activity measures

The NRFSs contain a specific module in which a questionnaire was used to collect information on the amount of physical activity performed by the selected individual. This module differentiates between intense, moderate, and walking physical activities, following the criteria established in the International Physical Activity Questionnaire (IPAQ), disaggregating these in turn into three levels of physical activity. The questions asked were the following:

1. For the intense level:
  - “In the last week, how many days did you do intense physical activities for at least 10 minutes?”
  - “Intense physical activity time in minutes?”
2. For the moderate level:
  - “In the last week, how many days did you do moderate physical activities for at least 10 min?”
  - “Moderate physical activity time in minutes?”
3. And for the low level:
  - “In the last week, how many days did you walk for at least 10 min?”
  - “Walked time in minutes?”

### 2.2. Descriptive statistics

Statistics reported in Table 1 were weighted by the corresponding expansion factor provided by the NRFSs. In general, the descriptive statistics do not show a significant variation between the NRFS samples except for the income and the level of physical activity. It is observed that the average age is between 43 and 45 years and that about 57% of the respondents are women. Concerning the marital status, about 55% of the individuals are married, 25% are single, between 10 and 11% are divorced, and 10% are widowed individuals. Regarding the employment situation, of the total number of respondents, it turned out that about 63% participate in the labor market, while about 37% remain inactive or unemployed. Income reports were deflated to 2005 through the alternative Bevacqua Consumer Price Index (CPI), re-scaled into four categories to match their ranks. The income reports were then converted to international dollars (I$), using the purchasing power parity conversion factor for 2014 equivalent to 4.65 Argentine pesos for international comparability (20). It is found that the highest proportion of people surveyed (61%) in the NRFS 2005 declared an average salary between I$ 1–200, 24% between I$ 201–400, 11% between I$ 401–600, and 4% of more than I$ 600. While in the NRFS 2009, 42% reported salaries between I$ 1–200, 32% between I$ 201–400, 17% between I$ 401–600, and 9% of more than I$ 600. Finally, in the NRFS 2013, 34% responded earning between I$ 1–200, 40% between I$ 201–400, 16% between I$ 401–600, and 10% more than I$ 600. Regarding education, it was broken down by individuals with and without a university degree, with the former accounting for around 14% of individuals and the latter around 86%. The health status variable was obtained through the self-reporting of the subjective perception of the health of the respondents, who were given the following question: “In general, how would you say your health is?” The response was distributed in 5 categories: excellent (NRFS 2005 = 8%; 2009 = 9%; 2013 = 11%), very good (NRFS 2005 = 24%; 2009 = 25%; 2013 = 23%), good (NRFS 2005 = 43%; 2009 = 43%; 2013 = 43%), regular (NRFS 2005 = 19%; 2009 = 18%; 2013 = 20%), and poor (NRFS 2005 = 3%; 2009 = 2%; 2013 = 3%). As for the households reporting children, it is observed that they vary from 36% to 39%. To approximate a measure for being overweight or obese, BMI is used, which reports an average value between 25 and 26 (being outside the normal weight range ≤ 24.9). Finally and related to the level of physical activity, it was observed that the low level increased markedly from the NRFS 2005 (45%) to the last two NRFSs (2009 = 56%; 2013 = 54%), with a decrease in individuals reporting a moderate level (from 45 to 31%), while the intense level remained relatively stable (from 10 to 14%).

**Table 1:** Descriptive statistics.

**Variables**	**NRFS 2005**		**NRFS 2009**		**NRFS 2013**	
	**Mean**	**SD**	**Mean**	**SD**	**Mean**	**SD**
Age	43.92	17.66	44.57	17.84	44.60	17.85
**Sex**						
Male	0.43	0.49	0.43	0.49	0.44	0.50
Female	0.57	0.49	0.57	0.49	0.56	0.50
**Income**						
I$1–200	0.61	0.006	0.42	0.004	0.34	0.005
I$201–400	0.24	0.006	0.32	0.004	0.40	0.005
I$401–600	0.11	0.004	0.17	0.003	0.16	0.004
I$>600	0.04	0.002	0.09	0.003	0.10	0.003
**Marital status**						
Married	0.54	0.50	0.55	0.50	0.53	0.50
Divorced	0.10	0.30	0.10	0.30	0.11	0.31
Widowed	0.10	0.29	0.10	0.29	0.10	0.29
Single	0.26	0.44	0.25	0.44	0.26	0.44
**Employment situation**						
Employed	0.63	0.48	0.62	0.48	0.62	0.49
Unemployed	0.37	0.48	0.38	0.48	0.38	0.49
**Educational level**						
Without university degree	0.86	0.48	0.86	0.43	0.86	0.49
With university degree	0.14	0.34	0.14	0.35	0.14	0.36
**Self-reported health**						
Excellent	0.08	0.28	0.09	0.29	0.11	0.32
Very good	0.24	0.43	0.25	0.43	0.23	0.42
Good	0.43	0.49	0.43	0.49	0.43	0.49
Regular	0.19	0.39	0.18	0.39	0.20	0.40
Poor	0.02	0.16	0.02	0.14	0.03	0.17
Child possession	0.39	0.49	0.38	0.48	0.36	0.48
BMI	25.49	4.41	26.01	4.50	26.44	4.60
Temperature (°C)	12.26	4.16	19.05	3.07	18.93	3.06
Rainfall (mm)	50.02	27.43	78.06	39.42	76.33	39.24
**Physical activity level**						
Intense	0.10	0.30	0.13	0.34	0.14	0.34
Moderate	0.44	0.49	0.32	0.46	0.31	0.46
Low	0.46	0.49	0.55	0.49	0.55	0.49

Continuous variables are expressed as a percentage and dummy variables as a proportion. All descriptive statistics were weighted by the corresponding expansion factor provided by the NRFSs. SD, standard deviation.

## 3. Econometric modeling

### 3.1. Pooled estimation

#### 3.1.1. Estimation by ordinary least squares

This work's main objective is to measure how the variables mentioned previously impact physical activity consumption. To do this, initially, we started by pooling the three NRFSs (2005, 2009, 2013) and by using a regression model estimated by ordinary least squares (OLS), which can be described as follows:

(1) $$\begin{array}{l} ln\left(Y_{i}\right)=\beta {\text{X}}_{i}+\epsilon_{i},i=1,...,I \end{array}$$

In Equation (1), two dependent variables are used (the descomposition of the outcome variables can be seen in Supplementary Table 2). The first outcome variable is computed as $\Omega_{i}=\frac{\overset{N}{\underset{j=1}{\sum }}\theta_{i,j}}{N}$, where θ_*i,j*_ are the minutes of physical activity consumed by the individual *i* at an intensity *j*, which represents the simple average of the minutes consumed in the week before the survey of intense, moderate, and low physical activities. Then, $\Omega_{i}^{p}=\frac{\overset{N}{\underset{j=1}{\sum }}\theta_{i,j}\times \psi_{i,j}}{{\text{Ψ}}_{i}}$ is used as a dependent variable, where θ_*i,j*_ is weighted by ψ_*i,j*_ which corresponds to the weighting applied for each individual *i* at each intensity *j*, allowing one to incorporate a greater weight to the minutes of physical activity that are carried out at a higher intensity. These two dependent variables are represented by *ln*(*Y*_*i*_), which takes the logarithm of the previously constructed variables to normalize the values of the distribution. The vector **X**_*i*_ represents the independent variables of interest for each individual *i* and is composed of continuous variables such as income, age, BMI, temperature and rainfall, and dummy variables such as gender, marital status, educational level, employment status, child possession, self-reported health status, geographic regions, and survey period. Finally, ϵ_*i*_ represents the classic error term.

#### 3.1.2. Estimation by Heckman two-stage model

The OLS was used to previously only recognizes individuals who decided to participate in the consumption of physical activity. Another alternative is to use a two-stage econometric model, where the factors that influence the participation decision can be differentiated from those associated with the amount consumed after the individual decided to participate. In this sense, the method developed by Heckman (21, 22) is used, allowing one to solve the problem of sample selectivity. If all the individuals in the sample decided to participate in the consumption of physical activity, the following would be given:

(2) $$\begin{array}{l} E\left[Y_{i}\right]=\beta {\text{X}}_{i} \end{array}$$

But if for some individuals *Y*_*i*_ = 0, the expected value of the amount of physical activity consumed would be:

(3) $$\begin{array}{l} E\left[Y_{i}\right]=Prob\left(Y_{i}>0\right)\cdot E\left[Y_{i}|Y_{i}>0\right]+Prob\left(Y_{i}\leq 0\right)\cdot 0 \end{array}$$

Therefore, when applying the Heckman procedure, two decisions are recognized. The first refers to the individual's decision to participate in the consumption of physical activity and the second to the decision about the amount allocated to said consumption, which is conditioned by the first decision. For this, two vectors of variables (**X**_*i*1_, **X**_*i*2_) are used where **X**_*i*1_ affects the decision to participate and **X**_*i*2_ the amount of consumption. Therefore, in order to deal with the problem of incidental truncation, a selection equation is added:

(4) $$\begin{array}{l} s_{i}^{*}=\alpha {\text{X}}_{i2}+v_{i} \end{array}$$

where $s_{i}^{*}$ is a latent unobservable variable that determines the probability of participation in the consumption of physical activity for each individual *i*, **X**_*i*2_ represents a vector of independent terms, and *v*_*i*_ ~ *Normal*(0, 1) is the error term that is normally distributed. Since we did not observe $s_{i}^{*}$ directly, the binary variable *s*_*i*_ is captured by the following mechanism:

(5) $$\begin{array}{l} s_{i}=\{\begin{array}{ccc} 1 & \text{si} & s_{i}^{*}>0\\ 0 & \text{si} & s_{i}^{*}\leq 0 \end{array} \end{array}$$

Therefore, *Y*_*i*_ is observed when *s*_*i*_ = 1. According to Equation (5), the probability of the response of *s*_*i*_ can be derived as:

(6) $$\begin{array}{l} \begin{array}{l} P\left(s_{i}=1|{\text{X}}_{i2}\right)=P\left(s_{i}^{*}>0|{\text{X}}_{i2}\right)=P\left({\text{X}}_{i2}\alpha +v_{i}>0|{\text{X}}_{i2}\right)\\ \;=P\left(v_{i}>-{\text{X}}_{i2}\alpha |{\text{X}}_{i2}\right)\\ \;=1-\Phi \left(-{\text{X}}_{i2}\alpha \right)=\Phi \left({\text{X}}_{i2}\alpha \right) \end{array} \end{array}$$

Φ(·) is a function that takes values within the range of zero and one (0 < Φ(·) < 1), whose cumulative distribution of a normal random variable can be expressed as:

(7) $$\begin{array}{l} \Phi \left(z\right)=\int_{-\infty }^{z}\phi \left(v\right)dv \end{array}$$

Thus, the estimation sequence is performed by the Heckman procedure in two steps. The first is to estimate $\frac{\alpha }{\sigma_{v}}$ using a probit model, which is computed by maximizing the probability function:

(8) $$\begin{array}{l} \ell =\underset{i\in S_{2}}{\prod }\left[1-\Phi \left(\frac{\alpha {\text{X}}_{i2}}{\sigma_{v}}\right)\right]\underset{i\in S_{1}}{\prod }\Phi \left(\frac{\alpha {\text{X}}_{i2}}{\sigma_{v}}\right) \end{array}$$

in relation to $\frac{\alpha }{\sigma_{v}}$, where σ_*v*_ is the variance of *v* and *S*_1_ is the set of individuals who decide to participate in the consumption of *Y*, while *S*_2_ is the set of individuals who do not decide to participate in said consumption. Then, to estimate $\frac{\alpha }{\sigma_{v}}$, the function *h*(·) is added to Equation (8), leaving the equation of the quantity consumed expanded:

(9) $$\begin{array}{l} Y_{i}=\beta {\text{X}}_{i1}+\frac{\sigma_{\epsilon v}}{\sigma_{v}}h\left(\frac{\alpha {\text{X}}_{i2}}{\sigma_{v}}\right)+\epsilon_{i} \end{array}$$

estimated by OLS. Consequently, the parameter estimates will be unbiased and consistent as they are corrected for selectivity.

### 3.2. Pseudo panel estimation

As in most developing countries, longitudinal data are not available in Argentina because of the high cost of following a set of individuals over a given period. Therefore, in this paper, we chose to follow the strategy of Deaton (13), developing (based on the use of repeated cross-sectional data) a series of fictitious cohorts to constitute a pseudo panel. This method allows one to take advantage of the benefits of working with panel data. Deaton's technique consists of constructing a series of fictitious cohorts and taking their average values. The cohort level model can be described as:

(10) $$\begin{array}{l} {\bar{Y}}_{c,t}=\beta {\bar{\text{X}}}_{c,t}+{\bar{\mu }}_{c,t}+{\bar{\epsilon }}_{c,t},c=1,\cdots \;,C;t=1,\cdots \;,T \end{array}$$

where ${\bar{Y}}_{c,t}$ is the dependent variable of the cohort *c* in the period *t*, ${\bar{\text{X}}}_{c,t}$ corresponds to the average of the values of the vector **X**_*i,t*_ for each cohort *c* in each period *t*, ${\bar{\mu }}_{c}$ is the fixed-effect of the cohort which remains constant since the cohorts are the same in the different periods, and ${\bar{\epsilon }}_{c,t}$ is the error term, which will not have the same value in each period since average cohort values are computed for different sets of individuals. To solve this measurement error, several authors have proposed estimators called “Error in Variables” (13, 23). However, it is observed that in most empirical studies, estimates by fixed- and random-effects are used, claiming that the measurement error can be left out if the number of observations per cohort is large enough, assuming that ${\bar{\mu }}_{c,t}=\mu_{c}$ for all *t* (24). This way, the estimated model would be:

(11) $$\begin{array}{l} {\bar{Y}}_{c,t}=\beta {\bar{\text{X}}}_{c,t}+{\bar{\mu }}_{c}+{\bar{\epsilon }}_{c,t},c=1,\cdots \;,C;t=1,\cdots \;,T \end{array}$$

As mentioned earlier, a worrying issue for the correct interpretation of the coefficients is the inverse causality between the dependent variable and the other covariates. For example, one would not be sure whether an individual's increased self-reported health leads to increased physical activity consumption or whether the change in physical activity consumption leads to an increase/decrease in health. To address this problem, it is proposed to use the following model:

(12) $$\begin{array}{r} Y_{c,t+1}^{T}=\beta_{1}ln\left(inc_{c,t}\right)+\beta_{2}ln\left(Y_{c,t}\right)+\beta_{k}{\text{X}}_{c,t}+\mu_{c}+\epsilon_{c,t},\\ c=1,\cdots \;,C;t=1,\cdots \;,T \end{array}$$

Here, $Y_{c,t+1}^{T}$ is the future relative variation rate of the cohort *c* in the period *t* + 1. This rate is calculated for minutes of physical activity unweighted by its intensity as $\Phi_{c,t+1}=\frac{\overset{N}{\underset{j=1}{\sum }}\theta_{c,j,t+1}-\overset{N}{\underset{j=1}{\sum }}\theta_{c,j,t}}{\Theta_{c,t}}$, where the sum of the minutes of physical activity in the cohort *c* in the period *t* + 1 is subtracted from the same cohort of the previous period *t*, dividing the result by the sum of the minutes of the period *t*. Then, the rate of minutes weighted by the intensity is computed as $\Phi_{c,t+1}^{p}=\frac{\overset{N}{\underset{j=1}{\sum }}\theta_{c,j,t+1}\times \psi_{c,j,t+1}-\overset{N}{\underset{j=1}{\sum }}\theta_{c,j,t}\times \psi_{c,j,t}}{\overset{N}{\underset{j=1}{\sum }}\theta_{c,j,t}\times \psi_{c,j,t}}$, where the minutes of physical activity are weighted by ψ representing the value of the METs for each intensity. *ln*(*inc*)_*c,t*_ is the logarithm of the income of the cohort *c* in the period *t*, and *ln*(*Y*)_*c,t*_ is the logarithm representing the amount of physical activity (Ω_*c,t*_ or $\Omega_{c,t}^{p}$) corresponding to the cohort *c* in the period *t*, which is similar to the dependent variables used in the previous estimates with the difference being that it is calculated here at the cohort level instead of at the individual level. **X**_*c,t*_ is a vector of control regressors of the cohort *c* in the current period *t*. Note that, from an econometric point of view, using the rate of variation of the period *t* + 1 ($Y_{c,t+1}^{T}$) reduces the simultaneity bias between physical activity consumption and the variables of interest. In this sense, it is difficult to think that future variation in physical activity consumption has any influence on, for example, present health.

Two techniques are used to estimate this model for panel data, the first of which is a fixed-effect estimation. In Equation (12), it is considered that there are unobserved factors that vary over time and others that are constant but that affect physical activity consumption. The fixed-effects method allows μ_*c*_ to capture all unobserved effects that affect physical activity consumption and do not vary over time. In this case, the unobserved effects refer to each of the cohorts. The term ϵ_*c,t*_ represents unobserved effects that change over time and affect physical activity consumption. One of the advantages of using the fixed-effects model is that it allows μ_*c*_ to be correlated with *ln*(*inc*)_*c,t*_, *ln*(*Y*)_*c,t*_ and **X**_*c,t*_, allowing the problem of unobserved heterogeneity to be solved. In order to eliminate from the equation the unobserved effects in the estimation by fixed-effects, a transformation of the variables is carried out. First, the mean values of the variables must be estimated, and the following equation is obtained:

(13) $$\begin{array}{l} {\bar{Y}}_{c,t+1}^{T}=\beta_{1}\bar{ln\left(inc_{c,t}\right)}+\beta_{2}\bar{ln\left(Y_{c,t}\right)}+\beta_{k}{\bar{\text{X}}}_{c,t}+\mu_{c}+{\bar{\epsilon }}_{c} \end{array}$$

Then, Equation (12) is subtracted from Equation (13) and obtains:

(14) $$\begin{array}{l} {\ddot{Y}}_{c,t+1}^{T}=\beta_{1}\ddot{ln\left(inc_{c,t}\right)}+\beta_{2}ln\left({\ddot{Y}}_{c,t}\right)+\beta_{k}{\ddot{\text{X}}}_{c,t}+{\ddot{\epsilon }}_{c,t} \end{array}$$

where the variables are expressed in units of deviations from their mean, thus eliminating unobserved effects, to then perform the regression by OLS.

The second strategy used was random-effects estimation. One of the main differences with the fixed-effects estimator is that when using this model, it must be assumed that μ_*c*_ is not correlated with the explanatory variables in each of the periods. However, the advantage of using this estimation method, as long as the previous assumption is respected, is that efficient estimators will be obtained, which is not the case with fixed-effect estimation, given that the transformation used to eliminate unobserved effects causes the estimators to be inefficient. The random-effects model is described as:

(15) $$\begin{array}{l} Y_{c,t+1}^{T}=\beta_{1}ln\left(inc_{c,t}\right)+\beta_{2}ln\left(Y_{c,t}\right)+\beta_{k}{\text{X}}_{c,t}+\nu_{c,t} \end{array}$$

with ν_*c,t*_ = μ_*c*_ + ϵ_*c,t*_. This model is estimated by generalized least squares to control the autocorrelation of the error terms (25).

To decide which of the models is the most appropriate, the Hausman test was used to check whether there are systematic differences between the model estimates.

## 4. Results and discussion

### 4.1. Pooled estimation

The estimates by OLS, and the Heckman two-stage estimation can be found in Tables 2, 3. Each of them contains estimates of the logarithm of Ω_*i*_ and $\Omega_{i}^{p}$ at the aggregate level and are stratified by gender.

**Table 2:** Estimation by ordinary least squares.

	**Simple average [*****Ln***(Ω_*****i*****_)**]**			**Weighted average [** $Ln\left(\Omega_{i}^{p}\right)$ **]**		
**Variables**	**Overall**	**Female**	**Male**	**Overall**	**Female**	**Male**
Ln (income)	-0.026^***^	-0.029^**^	-0.028^*^	0.004	0.003	-0.006
	(0.010)	(0.013)	(0.014)	(0.010)	(0.014)	(0.016)
Age	-0.108^***^	-0.062^***^	-0.154^***^	-0.113^***^	-0.058^***^	-0.172^***^
	(0.009)	(0.011)	(0.013)	(0.009)	(0.012)	(0.014)
Age^2^	0.002^***^	0.002^***^	0.003^***^	0.002^***^	0.002^***^	0.003^***^
	(0.000)	(0.000)	(0.000)	(0.000)	(0.000)	(0.000)
Age^3^	-0.000^***^	-0.000^***^	-0.000^***^	-0.000^***^	-0.000^***^	-0.000^***^
	(0.000)	(0.000)	(0.000)	(0.000)	(0.000)	(0.000)
Sex (female = 1)	-0.164^***^			-0.309^***^		
	(0.015)			(0.016)		
**Marital status**						
Divorced	0.072^***^	0.034	0.106^**^	0.103^***^	0.049	0.149^***^
	(0.027)	(0.035)	(0.044)	(0.029)	(0.037)	(0.047)
Widowed	0.037	0.052	0.017	0.088^***^	0.050	0.041
	(0.030)	(0.037)	(0.058)	(0.032)	(0.039)	(0.063)
Single	0.116^***^	0.056^*^	0.191^***^	0.158^***^	0.076^**^	0.234^***^
	(0.023)	(0.032)	(0.035)	(0.025)	(0.033)	(0.038)
**Educational level**						
High school	0.088^***^	0.080^***^	0.110^***^	0.116^***^	0.110^***^	0.146^***^
	(0.016)	(0.022)	(0.024)	(0.017)	(0.023)	(0.026)
University degree	0.138^***^	0.069^**^	0.241^***^	0.214^***^	0.149^***^	0.325^***^
	(0.022)	(0.029)	(0.035)	(0.024)	(0.030)	(0.038)
**Employment situation**						
Employed	0.197^***^	0.203^***^	0.242^***^	0.235^***^	0.226^***^	0.308^***^
	(0.018)	(0.022)	(0.032)	(0.019)	(0.023)	(0.035)
Unemployed	0.307^***^	0.393^***^	0.227^***^	0.317^***^	0.417^***^	0.239^***^
	(0.036)	(0.045)	(0.060)	(0.038)	(0.048)	(0.065)
Child possession	-0.085^***^	-0.086^***^	-0.049	-0.092^***^	-0.104^***^	-0.046
	(0.021)	(0.029)	(0.030)	(0.022)	(0.031)	(0.033)
**Self-reported health**						
Regular	0.750^***^	0.729^***^	0.788^***^	0.746^***^	0.725^***^	0.789^***^
	(0.046)	(0.057)	(0.077)	(0.049)	(0.060)	(0.083)
Good	1.059^***^	1.023^***^	1.136^***^	1.077^***^	1.034^***^	1.176^***^
	(0.045)	(0.056)	(0.075)	(0.048)	(0.059)	(0.082)
Very good	1.220^***^	1.185^***^	1.294^***^	1.299^***^	1.245^***^	1.403^***^
	(0.047)	(0.058)	(0.078)	(0.050)	(0.061)	(0.084)
Excelent	1.396^***^	1.306^***^	1.519^***^	1.526^***^	1.415^***^	1.678^***^
	(0.050)	(0.063)	(0.081)	(0.053)	(0.066)	(0.087)
BMI	-0.024^***^	-0.023^***^	-0.026^***^	-0.027^***^	-0.027^***^	-0.031^***^
	(0.002)	(0.002)	(0.003)	(0.002)	(0.002)	(0.003)
Observations	91,678	51,261	40,417	91,678	51,261	40,417
R-squared	0.085	0.090	0.080	0.107	0.103	0.108

Robust standard errors in parentheses.

*** *p* < 0.01,

** *p* < 0.05,

* *p* < 0.1.

Reference categories: Sex (male), Marital status (married), Educational level (incomplete high school), Employment situation (inactive), Self-reported health (poor). All estimates are controlled by climate variables (mean temperature and rainfall), and dummies for region and year.

**Table 3:** Estimation by Heckman two-stage model.

	**Simple average [*****Ln***(Ω_*****i*****_)**]**			**Weighted average [** $Ln\left(\Omega_{i}^{p}\right)$ **]**		
**Variables**	**Overall**	**Female**	**Male**	**Overall**	**Female**	**Male**
Ln (income)	-0.039^***^	-0.040^***^	-0.041^***^	-0.002	0.001	-0.015
	(0.005)	(0.007)	(0.008)	(0.007)	(0.009)	(0.010)
Age	-0.013^*^	0.014	-0.055^***^	-0.025^**^	0.015	-0.069^***^
	(0.008)	(0.010)	(0.012)	(0.010)	(0.013)	(0.015)
Age^2^	0.000^**^	-0.000	0.001^***^	0.000^**^	-0.000	0.001^***^
	(0.000)	(0.000)	(0.000)	(0.000)	(0.000)	(0.000)
Age^3^	-0.000^***^	-0.000	-0.000^***^	-0.000^***^	0.000	-0.000^***^
	(0.000)	(0.000)	(0.000)	(0.000)	(0.000)	(0.000)
Sex (female=1)	-0.118^***^			-0.303^***^		
	(0.009)			(0.012)		
**Marital status**						
Divorced	0.053^***^	0.009	0.110^***^	0.091^***^	0.029	0.160^***^
	(0.015)	(0.020)	(0.024)	(0.020)	(0.026)	(0.032)
Widowed	0.029^*^	0.005	0.048	0.091^***^	0.006	0.077^*^
	(0.018)	(0.022)	(0.033)	(0.023)	(0.029)	(0.044)
Single	0.034^**^	-0.010	0.092^***^	0.081^***^	0.013	0.132^***^
	(0.014)	(0.018)	(0.021)	(0.018)	(0.024)	(0.028)
**Educational level**						
High school	-0.070^***^	-0.039^**^	-0.079^***^	-0.031^*^	-0.000	-0.045^*^
	(0.012)	(0.015)	(0.019)	(0.016)	(0.020)	(0.025)
University degree	-0.149^***^	-0.121^***^	-0.145^***^	-0.053^**^	-0.024	-0.068
	(0.019)	(0.021)	(0.035)	(0.025)	(0.027)	(0.046)
**Employment situation**						
Employed	0.131^***^	0.100^***^	0.214^***^	0.188^***^	0.131^***^	0.303^***^
	(0.011)	(0.016)	(0.018)	(0.014)	(0.020)	(0.024)
Unemployed	0.063^***^	0.061	0.103^***^	0.092^***^	0.092^*^	0.122^***^
	(0.025)	(0.037)	(0.034)	(0.032)	(0.048)	(0.045)
Child possession	0.018	-0.004	0.030^*^	0.008	-0.020	0.038
	(0.013)	(0.018)	(0.018)	(0.017)	(0.023)	(0.023)
**Self-reported health**						
Regular	0.063	0.056	0.129	0.137^*^	0.093	0.160
	(0.054)	(0.072)	(0.081)	(0.071)	(0.093)	(0.106)
Good	0.093	0.077	0.198^**^	0.223^**^	0.144	0.287^**^
	(0.069)	(0.092)	(0.101)	(0.090)	(0.120)	(0.132)
Very good	0.136^*^	0.122	0.248^**^	0.339^***^	0.245^*^	0.411^***^
	(0.076)	(0.102)	(0.110)	(0.099)	(0.133)	(0.144)
Excelent	0.213^***^	0.193^*^	0.341^***^	0.476^***^	0.374^***^	0.550^***^
	(0.082)	(0.108)	(0.121)	(0.108)	(0.140)	(0.158)
BMI	-0.007^***^	-0.006^***^	-0.009^***^	-0.012^***^	-0.011^***^	-0.015^***^
	(0.001)	(0.002)	(0.002)	(0.002)	(0.003)	(0.003)
Observations	91,678	51,261	40,417	91,678	51,261	40,417

Only Equation (9) is reported. Robust standard errors in parentheses.

*** *p* < 0.01,

** *p* < 0.05,

* *p* < 0.1.

Reference categories: Sex (male), Marital status (married), Educational level (incomplete high school), Employment situation (inactive), Self-reported health (poor). All estimates are controlled by climate variables (mean temperature and rainfall), and dummies for region and year.

For Ω_*i*_, the coefficient of the variable *ln*(*income*) has a negative sign and is significant at 1% (for OLS and Heckman models). These results suggest that higher levels of income increase the opportunity cost of time, therefore, in the face of a wage increase, the physical activity consumption decreases. However, these results should be interpreted with some caution because the income used corresponds to the household level. In addition, other factors may decrease the negative correlation between income and physical activity. For example, individuals with greater access to public goods that allow the consumption of physical activity may be an unobserved feature that could impact income and physical activity. Additionally, as higher incomes may be associated with more hours of work, an additional specification was estimated with the individuals who reported hours worked to assess whether the correlation between income and physical activity could be negative when capturing the temporary restriction of the individuals who work more hours. Given that the coefficient remained negative, it could be suggested that higher incomes (at the household level) predominate over those that can generate a negative correlation. Age, meanwhile, suggests a polynomial behavior, reducing the consumption of Ω_*i*_ with each additional year of age.

Gender is also significant. It is observed that women consume 17.82% (OLS) less Ω_*i*_. This can be explained due to the weight of household production on women (which is logical, but only for those who have families). Additionally, it is observed that single and divorced people increase the consumption of Ω_*i*_ compared to married people, but not widowed individuals. Educational level also plays an important role in the allocation of time to physical activities. Estimates by OLS report that individuals who have both high school and university degrees increase the consumption of Ω_*i*_ in relation to others. However, the Heckman model presents the first sign change in these coefficients. This may be because most educated people have more information about the risks of a sedentary lifestyle and education generally has high correlations with income, which is negatively related to Ω_*i*_. In addition, when household income is used, the education coefficient could be capturing the correlation between individual income and physical activity. Regarding the employment situation, it is observed that both unemployed and employed people consume more physical activity than inactive people. This makes sense since disaggregating the inactive variable finds a high proportion of individuals over 60, who for biological reasons are limited in physical activity consumption. It is also useful to remember that, as the economic theory points out, two possible effects can occur in the demand for physical activity, given an increase in the opportunity cost of time. The first is the substitution effect, which states that a higher opportunity cost of time depends on the hourly wage rate, so increasing this will increase the opportunity cost of any other non-work activity. However, the income effect can mitigate the previous one, because both active and non-active leisure are normal goods; with a higher income, the individual can demand more of these goods. In this case, there seems to be a greater predominance of the income effect; however, since the regressions are controlled by income, it can also be thought that the positive correlation between the employed person and the physical activity could capture some individual characteristic not observed. The variable of child possession reports a negative relationship being significant at the aggregate level, so an individual with at least one child consumes less Ω_*i*_ [−8.60% (OLS)].

The health report shows a significant impact on the consumption of physical activity. It is observed that individuals with a better self-perception of health consume more minutes of physical activity. The variable BMI is significant at 1% with a negative sign in both estimates at the aggregate level and by subgroups. However, it should be noted that these two variables can lead to endogeneity bias in the estimates associated with problems of inverse causality.

Finally, it should be noted that in the two-stage estimates using Heckman's procedure, no substantial changes are observed between the decision-to-participate equation (Supplementary Table 1) and the time-to-participate equation (Table 3). However, it should be mentioned that the education coefficients are positive for the decision to participate and negative for the time of participation. This may be because, as mentioned above, higher levels of education may influence people to decide to engage in physical activity, but if education is considered as an opportunity cost proxy, more educated people may see their time in physical activity reduced.

In relation to the estimates made on the consumption of physical activity weighted by their intensity $\Omega_{i}^{p}$, it is observed that both the signs and the statistical significance remain stable in almost all the variables. A first point to highlight is in the case of income. It is observed that this does not present statistical significance. It is worth clarifying that income does not escape suffering from endogeneity and inverse causality problems; consequently, this topic will be added to the next section analysis. Age presents a more marked (although small) decrease in Ω_*i*_ than in $\Omega_{i}^{p}$. This indicates the weight of the biological factors that limit the practice of more intense physical activities as age increases. Gender presents a significant variation to Ω_*i*_. It is observed that the coefficient of the dummy variable (1 = female) decreases much more than its base category (OLS = 36.21%). In relation to marital status, there is a greater increase in $\Omega_{i}^{p}$ than in Ω_*i*_ for single and divorced individuals with respect to those married. For the educational level, it is observed that $\Omega_{i}^{p}$ increases more than Ω_*i*_ in people who accredit both high school and university education in relation to the others. This is not surprising since the educational level, in addition to being a proxy for the opportunity cost, tends to increase the efficiency of health production of the most educated individuals, in part, because they have more information about the probability of obtaining greater benefits from more intense physical activities. Regarding the employment situation, it is observed that $\Omega_{i}^{p}$ is increased more than Ω_*i*_ for the employed individuals with respect to the unemployed. The child possession variable does not show a substantial variation between Ω_*i*_ and $\Omega_{i}^{p}$. The self-reported health coefficients remain significant, but the increases of $\Omega_{i}^{p}$ are higher compared to that of Ω_*i*_ for people who report better health levels. Meanwhile, it is observed that people with lower body mass indices consume more $\Omega_{i}^{p}$ than Ω_*i*_.

### 4.2. Pseudo panel estimation

The previous estimates can be questionable and difficult to interpret because even after controlling for all regressors, there may be problems of simultaneity between the consumption of physical activity and the variables of interest. Thus, we opted to build a pseudo panel through 18 cohorts, which is used to estimate Equation (13) where it approximates Ω_*c,t*_ and $\Omega_{c,t}^{p}$ through its relative variation rates over the previous period (Φ_*c,t*+1_ and $\Phi_{c,t+1}^{p}$). By using the future rate of relative variation, the bias is reduced by the inverse relationship and the dynamic dimension of the problem is recognized. In addition, the use of cohorts allows control by heterogeneity not observable between cohorts. The estimates for the period of *t* correspond to the NRFSs 2005 and 2009, while the NRFS 2013 is only used to calculate the future rate of variation.

**Figure 1:**
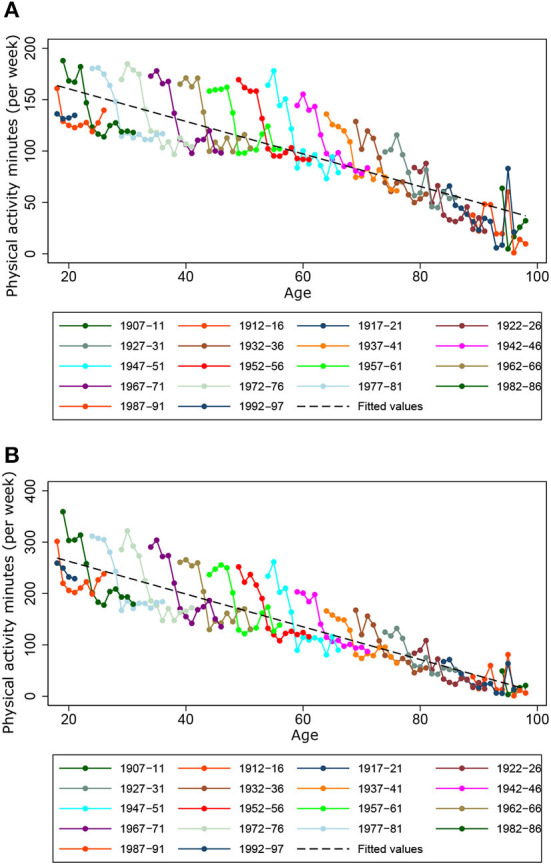
Average physical activity minutes per week. **(A)** Simple average. **(B)** Weighted average.

Figure 1 shows Ω_*c,t*_ (Figure 1A) and $\Omega_{c,t}^{p}$ (Figure 1B) disaggregated by cohort and age. The dashed lines correspond to the consumption of minutes of physical activity of each cohort, which contain the individuals from a younger to older age from left to right. Both the highest consumption peaks of Ω_*c,t*_ and $\Omega_{c,t}^{p}$ are located in the cohort 1982-1986. Additionally, it is observed that the slope of Figure 1A is less pronounced than that of Figure 1B; this shows that the consumption of $\Omega_{c,t}^{p}$ falls more with increasing age than the consumption of Ω_*c,t*_ does. These figures suggest that the increase in age (approximately with individuals older than 60 years) produces a marked decrease in the variance of $\Omega_{c,t}^{p}$ in relation to Ω_*c,t*_, which indicates, in this case, that the age factor is the one that takes the most force in the decision to perform physical activities at lower intensities. This can also be influenced by the retirement age cut-off where the opportunity cost of time decreases, causing a shift from more intense activities of shorter duration to less intense activities of longer duration.

The estimates by random-effects on the future relative variation rate (Φ_*c,t*+1_ and $\Phi_{c,t+1}^{p}$) associated with Ω_*c,t*_ and $\Omega_{c,t}^{p}$ are presented in Table 4, both at the aggregate level for women and men. It is observed that the coefficients of income, $\Omega_{c,t}^{p}$, age, gender, marital status, educational level, child possession, and self-reported health are statistically significant. The results suggest that both the present income and $\Omega_{c,t}^{p}$ negatively affect Φ_*c,t*+1_. This implies two issues, the first of which is that the rate of depreciation on health increases with age; consequently, as individuals age their health decreases directly impact physical activity consumption. Additionally, since physical activity has a delayed effect on health improvement [see Colman and Dave (26)], the individual may overestimate the present utility derived from past consumption and decrease future consumption of physical activity (e.g., past consumption of physical activity could improve one's self-perception of health because one's BMI decreased, but this situation could relax the consumption of physical activity in the future). The second issue is that there is a restriction in relation to the time available for physical activity consumption; therefore, by increasing the present consumption of physical activity, the available time stock is reduced, causing the marginal opportunity cost to be increasing, so it is logical to expect the future rate of variation to be a concave function of present consumption.

**Table 4:** Pseudo panel estimation.

	**Simple average (**Φ_*****c**, **t***+1**_**)**			**Weighted average (** $\Phi_{c,t+1}^{p}$ **)**		
**Variables**	**Overall**	**Female**	**Male**	**Overall**	**Female**	**Male**
Ln (income)	-0.795^***^	-0.648^*^	0.463^*^	-0.499^***^	-1.032^***^	0.155
	(0.226)	(0.366)	(0.265)	(0.221)	(0.370)	(0.304)
Ω_*c,t*_	-1.727^***^	-1.036^***^	-0.191			
	(0.274)	(0.361)	(0.331)			
$\Omega_{c,t}^{p}$				-1.312^***^	-1.041^***^	-0.783^***^
				(0.183)	(0.257)	(0.297)
Age	-0.021^**^	0.000	-0.010	-0.036^***^	-0.031^**^	-0.031^**^
	(0.009)	(0.017)	(0.011)	(0.010)	(0.015)	(0.014)
Sex (female)	-2.841^***^			-2.092^**^		
	(0.837)			(1.014)		
**Marital status**						
Divorced	1.304	1.287	0.221	0.396	3.201	1.515
	(0.919)	(2.698)	(2.044)	(1.171)	(2.684)	(2.629)
Single	-2.838^***^	-2.952^**^	-0.039	-1.465^**^	0.135	-1.821
	(0.447)	(1.146)	(1.103)	(0.589)	(0.928)	(1.286)
**Educational level**						
High school	-1.512^***^	-1.897	0.350	-1.355^**^	-2.058	-0.559
	(0.556)	(2.438)	(0.973)	(0.680)	(1.990)	(1.300)
University degree	-3.965^***^	-4.870	1.048	-3.066^*^	-1.556	-3.149
	(1.492)	(3.730)	(2.691)	(1.746)	(3.946)	(3.541)
**Employment situation**						
Employed	-0.078	-0.251	-0.119	-0.084	-0.217	-1.014
	(0.350)	(1.025)	(0.821)	(0.438)	(0.944)	(1.072)
Child possession	-1.580^***^	-2.752^**^	-0.339	-0.600	-2.962^***^	0.307
	(0.430)	(1.111)	(1.022)	(0.609)	(1.113)	(1.378)
**Self-perceived health**						
Very good	5.039^***^	8.98^***^	-2.128	2.741^***^	8.646^***^	-1.126
	(0.721)	(2.065)	(1.710)	(0.961)	(1.836)	(2.122)
BMI	0.062	0.223	-0.047	0.139	0.686^***^	-0.075
	(0.071)	(0.068)	(0.083)	(0.101)	(0.203)	(0.109)
Observations	33	33	33	33	33	33
Number of cohort	17	17	17	17	17	17

Robust standard errors in parentheses.

*** *p* < 0.01,

** *p* < 0.05,

* *p* < 0.1.

All estimates are controlled by climate variables (mean temperature and rainfall), and dummies for region and year.

Referring to the estimates on $\Phi_{c,t+1}^{p}$, it should be mentioned that both the present income and $\Omega_{c,t}^{p}$ impact negatively on $\Phi_{c,t+1}^{p}$. However, what is notable about these results is that the impact is slightly lower (approximately 37% for income and 24% for $\Phi_{c,t+1}^{p}$, in relation to Φ_*c,t*+1_) than the previous one. In the case of income, this difference could be explained by the high correlation between present and future income, which directly influences the opportunity cost of time, causing the individual to demand more intense activities of shorter duration. Likewise, it could be deduced that $\Omega_{c,t}^{p}$ impacts less on $\Phi_{c,t+1}^{p}$ due to, on the one hand, higher present demand for intense physical activities that generate greater future health benefits and since, on the other hand, the time restriction would be relaxed according to the previous argument.

## 5. Conclusions

It was noted that changes in the opportunity cost of time are highly significant and provide changes in individuals' decisions regarding the allocation of their time to active leisure activities. This is verified with the negative signs of the income coefficients in all the estimates and coincides with the results of Colman (26) and Humphreys (27). In addition, it is observed that when considering the intensity factor at which physical activities are consumed, income impacts less, suggesting that individuals faced with a wage increase reduce the time of consumption but increase its intensity.

When analyzing the employment situation between employed and inactive persons, it was observed that the former present greater consumption of physical activity than the latter. This is an exciting finding that indicates, using Becker's theory of time allocation (28), that the substitution effect produced by an increase in the wage rate is less than the income effect, and consequently, the amount of physical activity demanded (considering physical activity as a normal good) increases, generating a new equilibrium point with a higher consumption of physical activity than at the starting point. This may be an promising line of research for further work. Additionally, the increase in the coefficient of employed persons is greater when the intensity factor is considered, indicating that for employed individuals, a trade-off between time and intensity is generated.

In terms of educational level, the results indicate that individuals with more education consume less time in physical activity, which is reasonable because people with more education tend to have higher income levels; consequently, their opportunity cost of time is greater. The results of this study are also related to the economic model developed by Cawley (29), in which household production was included as a variable that could explain the variation in active leisure consumption. These results suggest that there is a strong effect of household production on women that decreases the consumption of physical activity, and it is verified with the negative coefficient of the gender variable and reinforced when the children coefficient is included, while the latter is not significant for men when subgroups disaggregate it. Regarding self-reported health and BMI, it was observed that people with higher health values consumed more physical activity and at a higher intensity, while the BMI presented a negative variation with respect to self-reported health.

Regarding dynamic estimates, for income, the results obtained are consistent with previous estimates for both Ω_*c,t*_ and $\Omega_{c,t}^{p}$. It indicates that the increase in present income generates a decrease in the future rate of relative variation. This makes sense since present income tends to have high correlations with future income, and therefore the increase in present income may impact the opportunity cost of time. On the other hand, both Ω_*c,t*_ and $\Omega_{c,t}^{p}$ negatively influence future consumption. This result indicates, as predicted by the Grossman (30) model, that the rate of health depreciation increases with age, and this directly influences the consumption of physical activity. Likewise, time constraint also plays an important role, since if an individual increases the present consumption of physical activity, they will have less time for future consumption; therefore, the marginal rate of inter-temporal substitution will tend to increase.

It is also important to mention some limitations of this study. It is likely that the future rate of relative variation (Φ_*c,t*+1_ or $\Phi_{c,t+1}^{p}$) and its present consumption [(*ln*(Ω_*c,t*_) or $ln\left(\Omega_{c,t}^{p}\right)$)] are jointly influenced by unobservable factors (e.g., cohort habits) that are captured in the error term. To the extent that these unobserved factors are correlated together, the β_2_ coefficient is biased. This endogeneity bias can lead to estimation problems and affects β_1_. A possible solution to the problem could be to use as an instrument of *ln*(Ω_*c,t*_) its lagging value [(*ln*(Ω_*c,t*−1_))]. However, this is not possible in the current context because by using pseudo panels, the observations would be too reduced, making this treatment impossible. Therefore, it should be noted that due to the lack of a suitable instrument, both the estimates of β_1_ and β_2_ lack causal interpretation, reflecting only conditional associations. Finally, our sample can only identify households in urban areas of at least 5,000 inhabitants, which means that our estimates lack external validity for areas other than those mentioned above.

However, these results may be useful in order to suggest some tools for the design of interventions that are aimed at increasing participation in physical activities. Since income is negatively related to physical activity consumption, one measure that could reverse, or at least improve, this situation is to propose physical activity programs at the workplace. There is also a significant gap in relation to gender and physical activity consumption. This indicates that programs designed to promote physical activity consumption in the population must take this issue into account. An attractive option would be the incorporation of day-care centers or family activities in sports clubs, to reduce the impact of children on physical activity consumption, especially on weekends when it could be higher. In the case of human capital, it is observed that people with a higher level of education (both secondary and university) present a lower demand for physical activity. Consequently, the problem to be solved appears before one's university education. Health education programs in secondary schools that allow one to know the benefits of regular physical activity could be efficient measures to improve this situation. The incorporation of campaigns and programs for physical activity during leisure time should also be an important issue for the agendas of policymakers, since it is observed that inactive people consume less physical activity than employed people. Further, because in this segment of the population the opportunity cost of time is not the biggest problem, free activities could encourage such participation. However, it is important to take into account, on the one hand, the age of the population at which programs to promote physical activity during leisure time are targeted, since there is a marked decrease in physical activity consumption for older adult populations, and on the other hand, the season in which these plans take place in order to mitigate the impact of climate on the decrease in demand for physical activities.

## Data availability statement

The original contributions presented in the study are included in the article/Supplementary material, further inquiries can be directed to the corresponding author.

## Author contributions

CMG-W produced the entire manuscript.

## Conflict of interest

The author declares that the research was conducted in the absence of any commercial or financial relationships that could be construed as a potential conflict of interest.

## Publisher's note

All claims expressed in this article are solely those of the authors and do not necessarily represent those of their affiliated organizations, or those of the publisher, the editors and the reviewers. Any product that may be evaluated in this article, or claim that may be made by its manufacturer, is not guaranteed or endorsed by the publisher.
