# Supplementary material for: Contemporary and dynamic effects of socio-economic factors on physical (in)activity: Does intensity matter?
Source: Front Public Health. 2022 Oct 6;10:1016353. doi: 10.3389/fpubh.2022.1016353 (PMC9583268; doi:10.3389/fpubh.2022.1016353)
Supplement: Supplementary file 1 [file Data_Sheet_1.PDF]

Supplementary Material

1 SUPPLEMENTARY TABLES AND FIGURES

1.1 Figures

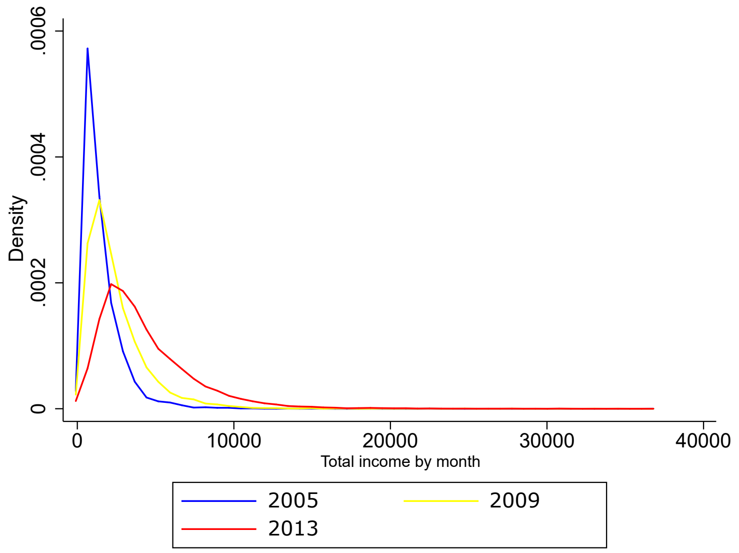

Figure S1. Monthly income deflated to 2005

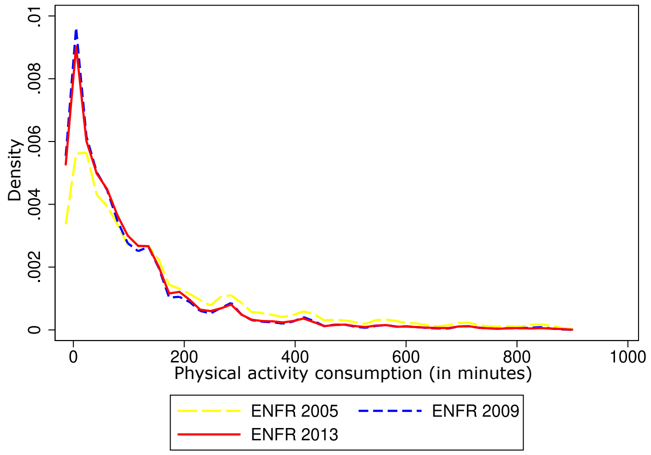

Figure 2a. Panel A: Simple average

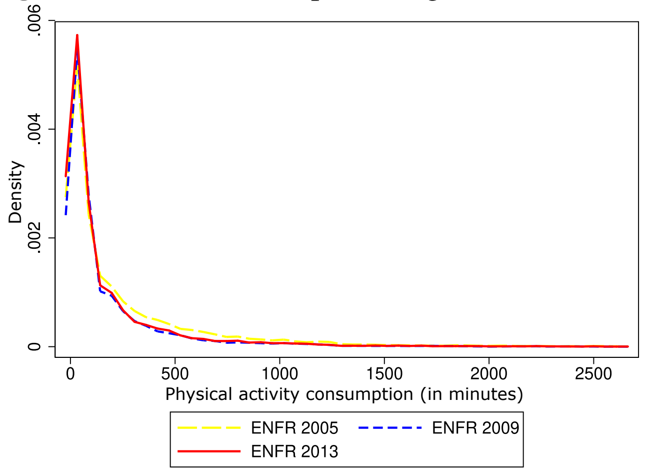

Figure 2b. Panel B: Weighted average

Figure 2. Average physical activity minutes per week

1.2 Tables

Table S1. Estimation by Heckman model (first-stage)

	Simple average ($Ln(\Omega_i)$)			Weighted average ($Ln(\Omega_i^p)$)		
Variables	Overall	Female	Male	Overall	Female	Male
Ln(income)	0.007	0.005	0.008	0.006	0.005	0.005
	(0.007)	(0.009)	(0.010)	(0.007)	(0.009)	(0.010)
Age	-0.074***	-0.050***	-0.101***	-0.074***	-0.050***	-0.102***
	(0.006)	(0.008)	(0.010)	(0.006)	(0.008)	(0.010)
Age^2	0.002***	0.001***	0.002***	0.002***	0.001***	0.002***
	(0.000)	(0.000)	(0.000)	(0.000)	(0.000)	(0.000)
Age^3	-0.000***	-0.000***	-0.000***	-0.000***	-0.000***	-0.000***
	(0.000)	(0.000)	(0.000)	(0.000)	(0.000)	(0.000)
Sex (female=1)	-0.056***			-0.056***		
	(0.010)			(0.010)		
**Marital status**						
Divorced	0.026	0.022	0.023	0.027	0.022	0.025
	(0.019)	(0.025)	(0.030)	(0.019)	(0.025)	(0.030)
Widowed	0.013	0.034	-0.010	0.012	0.035	-0.011
	(0.020)	(0.025)	(0.039)	(0.020)	(0.025)	(0.039)
Single	0.076***	0.053**	0.112***	0.076***	0.053**	0.111***
	(0.017)	(0.023)	(0.025)	(0.017)	(0.023)	(0.025)
**Educational level**						
High school	0.104***	0.080***	0.136***	0.104***	0.080***	0.135***
	(0.012)	(0.016)	(0.017)	(0.012)	(0.016)	(0.017)
University degree	0.192***	0.123***	0.290***	0.190***	0.122***	0.288***
	(0.016)	(0.021)	(0.026)	(0.016)	(0.021)	(0.026)
**Employment situation**						
Employed	0.061***	0.085***	0.045*	0.061***	0.084***	0.046**
	(0.012)	(0.015)	(0.023)	(0.012)	(0.015)	(0.023)
Unemployed	0.206***	0.273***	0.123***	0.200***	0.264***	0.121***
	(0.027)	(0.035)	(0.045)	(0.027)	(0.035)	(0.045)
Child possession	-0.071***	-0.063***	-0.050**	-0.069***	-0.063***	-0.047**
	(0.015)	(0.020)	(0.021)	(0.015)	(0.020)	(0.021)
**Self-reported health**						
Regular	0.423***	0.419***	0.436***	0.425***	0.423***	0.435***
	(0.029)	(0.036)	(0.048)	(0.029)	(0.036)	(0.048)
Good	0.611***	0.608***	0.640***	0.615***	0.612***	0.642***
	(0.029)	(0.036)	(0.047)	(0.029)	(0.036)	(0.047)
Very good	0.713***	0.709***	0.740***	0.718***	0.714***	0.742***
	(0.030)	(0.038)	(0.049)	(0.030)	(0.038)	(0.049)
Excelent	0.803***	0.759***	0.872***	0.807***	0.765***	0.873***
	(0.033)	(0.042)	(0.053)	(0.033)	(0.042)	(0.053)
BMI	-0.013***	-0.013***	-0.014***	-0.013***	-0.013***	-0.014***
	(0.001)	(0.001)	(0.002)	(0.001)	(0.001)	(0.002)
Observations	91,678	51,261	40,417	91,678	51,261	40,417

Note: Robust standard errors in parentheses. *** p<0.01, ** p<0.05, * p<0.1. Reference categories: Sex (male), Marital status (married), Educational level (incomplete high school), Employment situation (inactive), Self-reported health (poor). In these regressions, temperatures and rainfall were removed from the second stage to satisfy the exclusion restrictions. All estimates are controlled by dummies for region and year.

Table S2. Decomposition of $\Omega_{c,t}$, $\Omega^p_{c,t}$, $\Phi_{c,t+1}$, $\Phi^p_{c,t+1}$

Decomposition by individual	Decomposition by cohort
Physical activity minutes	
$\Theta_i = \sum_{j=1}^{N} \theta_{i,j}$	$\Theta_{c,t} = \sum_{j=1}^{N} \theta_{c,j,t}$
Physical activity intensity	
$\Psi_i = \sum_{j=1}^{N} \psi_{i,j}$	$\Psi_{c,t} = \sum_{j=1}^{N} \psi_{c,j,t}$
Average physical activity minutes	
$\Omega_i = \frac{\sum_{j=1}^{N} \theta_{i,j}}{N}$	$\Omega_{c,t} = \frac{\sum_{j=1}^{N} \theta_{c,j,t}}{N}$
Average physical activity minutes by intensity	
$\Omega^p_i = \frac{\sum_{j=1}^{N} \theta_{i,j} \times \psi_{i,j}}{\Psi_i}$	$\Omega^p_{c,t} = \frac{\sum_{j=1}^{N} \theta_{c,j,t} \times \psi_{c,j,t}}{\Psi_{c,t}}$
Future rate of relative variation	
$\Phi_{c,t+1} = \frac{\sum_{j=1}^{N} \theta_{c,j,t+1} - \sum_{j=1}^{N} \theta_{c,j,t}}{\Theta_{c,t}}$	$\Phi^p_{c,t+1} = \frac{\sum_{j=1}^{N} \theta_{c,j,t+1} \times \psi_{c,j,t+1} - \sum_{j=1}^{N} \theta_{c,j,t} \times \psi_{c,j,t}}{\sum_{j=1}^{N} \theta_{c,j,t} \times \psi_{c,j,t}}$

Note: j corresponds to each physical activity level (low, moderate, high). i corresponds to each individual. c corresponds to each cohort. t to each period. Ψ_i corresponds to the intensity (which is measured in METs and is a constant for each activity) of the consumption of each activity j for each individual i.
